# Effectiveness of lanreotide autogel 120 mg at extended dosing intervals for acromegaly

**DOI:** 10.1007/s12020-020-02424-z

**Published:** 2020-07-28

**Authors:** Ignacio Bernabéu, Carmen Fajardo, Mónica Marazuela, Fernando Cordido, Eva María Venegas, Pedro de Pablos-Velasco, Gonzalo Piedrola Maroto, María Pilar Olvera, Isabel Pavón de Paz, Davide Carvalho, Carme Romero, Guillermo De la Cruz, Cristina Álvarez Escolá

**Affiliations:** 1 Endocrinology and Nutrition Department, Hospital Clínico Universitario Santiago de Compostela, Santiago de Compostela, Spain; 2 Endocrinology and Nutrition Department, Hospital Universitario de La Ribera, Alzira (Valencia), Spain; 3 Endocrinology and Nutrition Department, Hospital Universitario La Princesa, Universidad Autónoma Madrid, Instituto Princesa, Madrid, Spain; 4 Faculty of Health Sciences and INIBIC, University of A Coruña, and Endocrinology and Nutrition Department, Complejo Hospitalario Universitario de A Coruña, A Coruña, Spain; 5 Endocrinology and Nutrition Department, Hospital Universitario Virgen del Rocío, Sevilla, Spain; 6 Endocrinology and Nutrition Department, University of Las Palmas de Gran Canaria. Spain, Las Palmas, Spain; 7 Endocrinology and Nutrition Departament Hospital Universitario Virgen de las Nieves, Granada, Spain; 8 Endocrinology and Nutrition Departament, Hospital Universitario Nuestra Señora de Candelaria, Santa Cruz de Tenerife, Spain; 9 Endocrinology and Nutrition Departament, Hospital Universitario de Getafe, Madrid, Spain; 10 Department of Endocrinology, Diabetes and Metabolism, Centro Hospitalar Universitário São João, Faculty of Medicine, i3S, Universidade do Porto, Porto, Portugal; 11 Adknoma Health Research S.L., Barcelona, Spain; 12 Ipsen Pharma, Barcelona, Spain; 13 Endocrinology and Nutrition Department, Hospital Universitario La Paz, Madrid, Spain

**Keywords:** Acromegaly, Lanreotide, Insulin-like growth factor 1, Somatostatin, Growth hormone

## Abstract

**Purpose:**

Recent data indicate that extended dosing intervals (EDIs) with lanreotide autogel 120 mg are effective and well-received among patients with acromegaly who have achieved biochemical control with monthly injections of long-acting somatostatin analogues (SSAs). We further evaluated the effectiveness of lanreotide autogel 120 mg delivered at EDIs (>4 weeks) in routine clinical practice.

**Methods:**

Cross-sectional, multicentre, observational study conducted to determine the effectiveness—measured by control of serum insulin-like growth factor 1 (IGF-1)—of lanreotide autogel 120 mg at dosing intervals >4 weeks for ≥6 months in selected patients with acromegaly treated in routine clinical practice (NCT02807233). Secondary assessments included control of growth hormone (GH) levels, treatment adherence, patient satisfaction, and quality of life (QoL) using validated questionnaires (EQ-5D, AcroQoL, and TSQM-9). Patients who received radiotherapy within the last 6 months were excluded.

**Results:**

Among 109 patients evaluated, mean (SD) age was 59.1 (13.2) years. IGF-1 values were normal (mean [SD]: 175.0 [74.5], 95% CI: 160.8 –189.1) in 91.7% of cases and normal in 91.4% of patients without previous radiotherapy treatment (*n* = 81). GH levels were ≤2.5 and ≤1 ng/mL, respectively, in 80.6% and 58.3%. Most patients were treated either every 5–6 (57.8%) or 7–8 weeks (38.5%), with 2.8% treated greater than every 8 weeks. The mean AcroQoL score was 63.0 (20.1). The mean global treatment satisfaction score (TSQM-9) was 75.1 (16.6). Treatment adherence (defined as no missed injections) was 94.5%.

**Conclusion:**

Lanreotide autogel 120 mg at intervals of >4 weeks provided IGF-1 control in more than 90% of patients with acromegaly. Treatment satisfaction and adherence were good. These findings support use of extended dosing intervals in patients who have achieved good biochemical control with long-acting SSAs.

## Introduction

Acromegaly is a rare endocrine disorder characterised by increased production of growth hormone (GH) and insulin-like growth factor 1 (IGF-1) due to a benign pituitary tumour [1, 2]. The mainstay of treatment is transsphenoidal surgery to resect or debulk the pituitary adenoma to normalise GH secretion [3]. Although surgery is curative in many cases, ~50% of patients require pharmacological therapy, usually consisting of long-acting somatostatin analogues (SSAs), generally either lanreotide autogel (Somatuline Depot in the United States) or octreotide long-acting release (LAR) [3–5]. The main aim of medical treatment is to normalise serum GH and IGF-1 levels in order to control symptoms and improve quality of life (QoL).

Conventional SSAs are generally injected on a monthly basis by healthcare professionals (HCPs) according to the drug label. However, given the chronic nature of acromegaly, the need for frequent visits to the clinic to receive injections can be burdensome for some patients and may negatively impact QoL, treatment adherence, and treatment satisfaction. The development of long-acting SSAs such as lanreotide autogel, which can be self-administered at home by the patient or a caregiver, represents a significant advance in the treatment of acromegaly [6, 7]. In addition, a growing body of evidence, including an expert consensus statement [4], suggests that extended dosing intervals (EDIs) with lanreotide autogel 120 mg of up to 8 weeks (versus the standard 4-week dosing interval) may be equally effective in patients who have achieved good biochemical control with long-acting SSAs [8–10]. The most relevant benefits of extending the treatment interval are fewer injections, lower costs, more convenient treatment; in addition, EDIs and/or dose reductions have both been shown to reduce the risk of treatment-related side-effects [3, 4, 9, 11]. Numerous open-label trials have shown that lanreotide autogel 120 mg administered at dosing intervals >4 weeks provides comparable biochemical control to that achieved with standard dosing intervals [4, 9–14]. Nevertheless, more data are needed to evaluate the effectiveness and safety of EDIs in routine clinical practice [11, 15].

In this context, the aim of the present cross-sectional, multicentre study was to determine the effectiveness, as measured by IGF-1 levels, of lanreotide autogel 120 mg administered at dosing intervals >4 weeks for more than 6 months in patients with acromegaly treated in routine clinical practice. Secondary aims were to evaluate patient satisfaction, treatment adherence, and QoL.

## Materials and methods

### Patients

Inclusion criteria were: (1) adults ≥18 years; (2) confirmed diagnosis of acromegaly; (3) under treatment with lanreotide autogel 120 mg at dosing intervals >4 weeks for ≥6 months before the study visit; (4) availability of recent GH and IGF-1 serum levels prior to the study visit; and (5) signed informed consent form.

Exclusion criteria were: (1) radiotherapy within the last 6 months; (2) active participation in another clinical trial; (3) presence of physical or mental disorders that could affect the participant’s capacity to sign the informed consent form; (4) pregnancy or breastfeeding; and (5) missing data with regard to the date lanreotide treatment was initiated or changes in the administration schedule during the prior 6 months.

### Design

SOMACROL was a multicentre, cross-sectional, observational study conducted in hospitals in Spain and Portugal (NCT02807233). The study included only a single visit designed to coincide with the patient’s follow-up consultation after ≥6 months of treatment with lanreotide autogel at dosing intervals >4 weeks. In accordance with routine clinical practice, the use of EDIs is limited to patients who have previously achieved clinical and biochemical control under the conventional dosing regimen (i.e., according to the drug label) [4]. Consequently, all patients in the present study presented normal IGF-1 levels at initiation of the EDI. Serum IGF-1 and GH levels were determined before the study visit.

The primary objective was to determine the effectiveness (control of serum IGF-1 levels) of lanreotide autogel 120 mg at EDIs (>4 weeks) administered in routine clinical practice in patients with acromegaly after at least 6 months of treatment. Secondary objectives were: (1) to assess treatment effectiveness based on control of serum GH levels; (2) to describe the schedule of lanreotide administration at EDIs in routine clinical practice; and (3) to evaluate QoL, patient satisfaction, and treatment adherence.

All patients who met the inclusion criteria and agreed to participate were enroled in the study after signing the informed consent form. At the single study visit, all data required to evaluate the study objectives were recorded on the electronic case report form (eCRF). At this visit, patients completed the following questionnaires: the generic health-related QoL measure EuroQoL-5D (EQ-5D) [16], the acromegaly-specific Acromegaly Quality of Life Questionnaire (AcroQoL) [17], and the Treatment Satisfaction Questionnaire for Medication (TSQM-9) [18]. Given that this was an observational study, all participants were treated in accordance with routine clinical practice and no additional evaluations or tests, apart from the aforementioned questionnaires, were requested.

The study was approved by the ethics committees at the participating hospitals. This study was conducted in accordance with the Declaration of Helsinki as revised in 2013 [19].

### Study variables and outcome measures

The main outcome measure was the serum IGF-1 level assessed immediately prior to the study visit, which was classified as normal or high by the treating physician based on the patient’s age and sex. Serum IGF-1 and GH levels were determined in local laboratories in accordance with the standard procedures in place at each participating centre. To rule out the possible influence of radiotherapy on the main endpoint, we separately assessed IGF-1 levels for the patients who did not receive radiotherapy.

Due to normal variations in routine clinical practice, a variety of dosing regimens (ranging from 5 to 8 weeks) were used. However, in accordance with the study inclusion criteria, the dosing interval was >4 weeks in all cases. For purposes of this study, patients were grouped according to the dosing intervals (5–6 weeks versus 7–8 weeks). The following dosing-related variables were evaluated: (1) proportion of patients treated at 5–6 or 7–8 week dosing intervals; (2) percentage of patients whose dosing regimen was adjusted <6 months before the study visit; (3) type (increase or decrease), reason, and date of adjustment in the dose or administration schedule; and (4) patient preference for self-administered versus HCP-administered injections.

The EQ-5D consists of a descriptive system to evaluate five different dimensions of QoL (mobility, self-care, usual activities, pain/discomfort, and anxiety/depression) and a self-rated visual analogue scale (VAS) ranging from 0 to 100, in which 0 is the worst and 100 is the best state of health imaginable [16]. AcroQoL provides a standardised score ranging from 0 (worst QoL) to 100 (best QoL) [17]. Treatment satisfaction scores using the TSQM-9 range from 0 (lower satisfaction) to 100 (higher satisfaction) [18].

Data on the dose, dose interval, and missed doses during the 6-month period before the study visit were retrospectively obtained from clinical records by the treating physician, who entered these data on the eCRF. The following dosing-related variables were recorded: (1) mean time (weeks) between injections; (2) number of injections received; (3) number of missed doses; (4) changes, if any, in the dose and/or dosing interval. Patients who missed ≥1 injection during the study period were considered non-adherent.

### Statistical analysis

Demographic and clinical data from the eCRFs were entered in a database created for this purpose. Descriptive analyses of all variables were performed. Categorical variables were summarised by absolute frequency (*n*) and percentage (%). Continuous variables were summarised by measures of central tendency and dispersion: mean, standard deviation (SD), 95% confidence interval (CI), median, and range.

The Kolmogorov–Smirnov test was used to determine data normality. The Student’s *t* test was used to evaluate normally-distributed continuous variables while the Mann–Whitney *U* test was used to evaluate variables with a non-normal distribution. Associations between the categorical variables were assessed with the Chi-square or Fisher’s exact test, as appropriate. SAS software (version 9.4; SAS Institute, Cary, NC) was used to perform all statistical analyses. Statistical significance was set at two-tailed *p* < 0.05.

The sample size calculation (*n* = 100) was based on an estimated prevalence of acromegaly in Spain of 60 cases per million [20], with 95% CI and 6% precision.

## Results

A total of 114 patients were recruited from 38 participating centres in Spain and a single centre in Portugal. Of these, five patients were excluded for failure to meet the study inclusion criteria, resulting in a total of 109 evaluable patients. The final sample consisted of 62 women (56.9%) and 47 men (43.1%). The mean age was 59.1 years (SD, 13.2). The sociodemographic and clinical characteristics of the patients are shown in Table 1.

**Table 1:** Sociodemographic and clinical characteristics^a^

Age, years	59.1 (13.2)
Women, *n* (%)	62 (56.9%)
Height, cm	165.3 (11.1)
Weight, kg	80.9 (19.1)
BMI, kg/m^2^	29.4 (5.3)
Comorbidities, *n* (%)^b^	91 (83.5%)
Diabetes	31 (34.4%)
Hypertension	58 (64.4%)
Cholelithiasis	15 (16.7%)
Apnoea	18 (20.0%)
Neuropathies	8 (8.9%)
Other	57 (63.3%)
Time elapsed since diagnosis, years	12.3 (9.6)
Previous treatments (surgery or radiotherapy), *n* (%)	
Surgery alone	53 (48.6)
Radiotherapy alone	5 (4.6)
Surgery + radiotherapy	23 (21.1)
Neither surgery nor radiotherapy	28 (25.7)
Time elapsed since tumour resection, years	12.8 (9.4)
Time elapsed since radiotherapy, years	17.9 (9.4)
Previous pharmacological treatment, *n* (%)	
Lanreotide (other formulations)	7 (6.4%)
Mean (SD) dose, mg	68.6 (37.6)
Octreotide	28 (25.7%)
Mean (SD) dose, mg	31.1 (30.2)
Pegvisomant	1 (0.92%)
Cabergoline	6 (5.5%)
Bromocriptine	1 (0.92)

^a^All data given as mean (SD; standard deviation) unless otherwise indicated

^b^Some patients had ≥1 comorbidity

SSAs were the first-line medical treatment for acromegaly or related symptoms in nearly all patients (*n* = 108; 99%). Most of these patients (*n* = 73; 67.0%) received lanreotide autogel 120 mg while 35 patients (32.1%) were prescribed other formulations of lanreotide (*n* = 7) or octreotide (*n* = 28). One patient received pegvisomant as first-line therapy (Table 1). In terms of pharmacological treatments received prior to lanreotide autogel, 36 patients received a total of 43 treatments (Table 1). Of the 109 evaluable patients, 84 (77.1%) were taking other medications concomitantly with lanreotide, primarily for the treatment of cardiovascular (40.9%), gastrointestinal tract, or metabolic disorders (24.2%). Thirteen patients (11.9%) received concomitant treatment with cabergoline (*n* = 8) or pegvisomant (*n* = 5).

### Effectiveness

Blood tests performed immediately prior to the study visit showed mean (SD) IGF-1 values of 175.0 (74.5) μg/L (95% CI, 160.8–189.1), with 91.7% (95% CI, 86.6–96.9%) of patients presenting normal IGF-1 values. In the subgroup of patients who did not receive radiotherapy (*n* = 81), the results were similar, with 74 of these 81 patients (91.4%) presenting normal IGF-1 values.

The mean (SD) GH values were 2.1 (5.1) ng/mL (95% CI, 1.13–3.08). Overall, 80.6% and 58.3% of the patients, respectively, presented GH levels ≤2.5 or ≤1 ng/mL (Table 2). The mean time from diagnosis to initiation of EDI was 9.2 years (SD, 8.8; 95% CI: 7.5–10.9). The most common dosing interval was every 5–6 (57.8%) versus every 7–8 weeks (38.5%); however, in three patients (2.8%), the dosing interval was greater than 8 weeks.

**Table 2:** IGF-1 and GH levels at the study visit in patients treated with lanreotide autogel 120 mg

	Number of patients (%)	Mean (SD)
IGF-1 (μg/L)	109 (100%)	175.0 (74.5); 95% CI: 160.80–89.10
IGF-1 values^a^		
Normal	100 (91.7%)	n/a
High	9 (8.3%)	
Time elapsed since the last dose of lanreotide autogel, weeks	101	7.6 (5.9)
Time elapsed from the blood test to the visit, days	109	22.7 (25.4)
GH (ng/mL)	108 (99.1%)	2.1 (5.1)
GH ≤ 2.5 ng/mL		
Yes	87 (80.6%)	n/a
No	21 (19.4%)	
GH ≤ 1.0 ng/mL		
Yes	63 (58.3%)	n/a
No	45 (41.7%)	
GH (ng/mL)		
≤1.0	63 (58.3%)	n/a
>1 and ≤ 2.5	24 (22.2%)	
>2.5	21 (19.4%)	
GH level (ng/mL) in patients with normal IGF-1 levels		
≤1.0	59 (59.6%)	n/a
>1.0–≤2.5	22 (22.2%)	
>2.5	18 (18.2%)	

*n/a* not applicable, *SD* standard deviation, *CI* confidence intervals

^a^IGF-1 levels were classified as normal or high by the treating physician based on the patient’s age and sex

The study medication was delivered by HCPs in 82.6% of cases and self-injected in the remaining 17.4% of cases. Patient preferences for the form of administration yielded the same result, with 82.6% preferring administration by an HCP.

Eight patients (7.3%) required treatment adjustment (change in dose or dosing interval). A total of ten treatment adjustments were made in these eight patients, as follows: increase (*n* = 5) or decrease (*n* = 3) in the dosing interval, or dose decrease (*n* = 2). Thus, 70% of treatment adjustments were performed to extend the dosing interval or to decrease the dose. The mean (SD) time from treatment initiation to treatment adjustment was 2.3 (2.5) years (95% CI, 0.52–4.04).

**Fig. 1:**
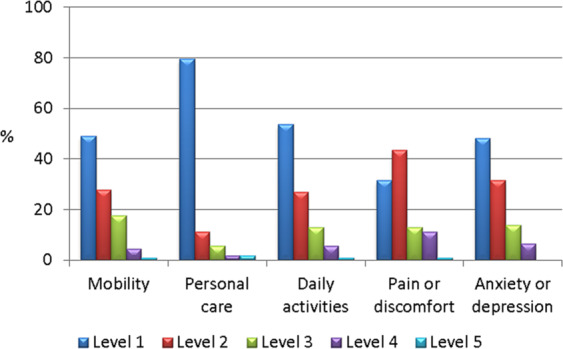
Quality of life. Results of the EQ-5D questionnaire. Levels 1–5 indicate, respectively, no, slight, moderate, severe or extreme problems

The most commonly reported issues on the EQ-5D questionnaire were pain/discomfort (68.5% of the respondents), followed by anxiety/depression (51.9%), and difficulties with mobility (50.9%), daily activities (46.3%), or self-care (20.4%) (Fig. 1). The mean (SD) EQ-5D VAS score was 69.7 (17.9).

**Fig. 2:**
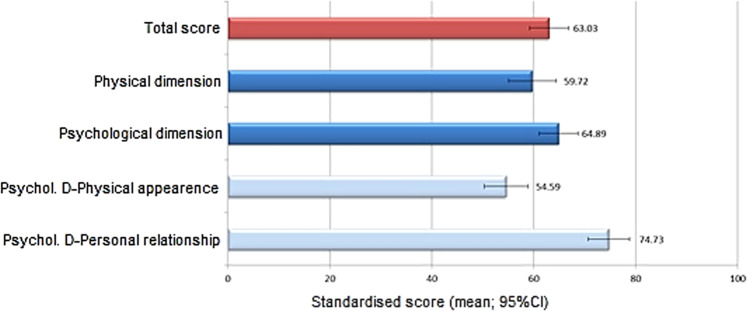
Quality of life. Results of the Acromegaly Quality of Life Questionnaire. Higher scores indicate better quality of life

The mean (SD) total standardised score on the AcroQoL questionnaire was 63.0 (20.1), ranging from 3.4 to 96.6 (Fig. 2). The mean scores on the physical and psychological dimensions of this scale were, respectively, 59.7 (24.5) and 64.9 (20.1). The results of the four standardised dimensions (physical, psychological, psychological-physical appearance, and psychological-interpersonal relationship) are shown in Fig. 2.

The mean (SD) scores on the TSQM-9 questionnaire were as follows: (1) Effectiveness: 70.6 (18.7); (2) Convenience: 69.1 (17.6); and (3) Global satisfaction: 75.1 (16.6).

### Treatment adherence

**Fig. 3:**
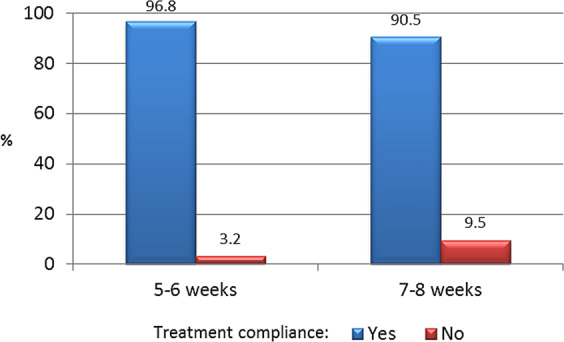
Treatment adherence according to dosing interval: 5–6 weeks versus 7–8 weeks. Patients who missed ≥1 injection were considered non-adherent

During the 6-month period prior to the study visit, the study participants received a mean of 3.9 (1.0) injections administered at 6 week intervals on average. Based on the eCRF data, 94.5% (*n* = 103) were considered treatment adherent (no missed injections) while 5.5% (*n* = 6) of patients missed one or more injections during the study period. Four patients discontinued treatment due to lack of efficacy (*n* = 1) or for unspecified reasons (*n* = 3). No statistically significant differences were observed in adherence rates between the patient group treated every 5–6 weeks versus those treated every 7–8 weeks (Fig. 3).

## Discussion

This study confirms the effectiveness of acromegaly treatment with lanreotide autogel 120 mg at EDIs > 4 weeks for at least 6 months in routine clinical practice, consistent with previous reports [4, 9–14]. The most common dosing intervals in our sample (95.4% of patients) were every 5–6 weeks (57.8%) or every 7–8 weeks (38.5%). Most patients (91.7%) maintained normal IGF-1 values and GH levels, which were ≤2.5 and ≤1 ng/mL in 80.6% and 58.3%, respectively. The results in the subgroup of non-irradiated patients (*n* = 81) were virtually identical to those in the main group, with 91.4% presenting normal IGF-1 levels.

### Effectiveness

The effectiveness of the EDI regimen in our sample is further supported by the finding that only three patients (2.8%) required a more frequent dosing interval. Indeed, most of the treatment adjustments (70%) were performed to extend the dosing interval or to decrease the dose. Importantly, only four patients discontinued treatment, only one due to “lack of efficacy”. One patient discontinued treatment—despite reporting symptom improvement—due to poor gastrointestinal tolerance.

There is a growing body of evidence to support EDIs in well-selected patients, with numerous open-label studies showing good symptom control with lanreotide autogel 120 mg administered at EDIs [4, 9–14]. Schopohl et al. found that switching to lanreotide autogel 120 mg at 6- or 8-week intervals provided hormonal control and QoL equivalent to that achieved with 10 or 20 mg of octreotide LAR administered every 4 weeks [9]. In the LEAD study [10], more than 75% of patients maintained IGF-1 control at 48 weeks after switching octreotide LAR 10 or 20 mg/monthly to lanreotide autogel at EDIs, while serum GH levels remained ≤2.5 ng/mL in 90% of the patients at both 24 and 48 weeks [10]. Our results, in a smaller but real-world, practice-based multicentre series, were similar.

The results of the LEAD study [10] suggest that the best candidates for lanreotide 120 mg at EDIs are those patients who have achieved clinical and biochemical disease control under the conventional dosing regimen (i.e., every 28 days with lanreotide autogel 120 mg). Patients who have not achieved sustained disease control under the conventional dosing schedule would be unlikely to benefit from EDIs [4].

A prospective, observational study conducted by Orlewska et al. [11] in Poland in a real-world sample of 143 patients with acromegaly found that 50% of the patients were receiving lanreotide autogel 120 mg at dosing intervals >4 weeks, a finding that provides further evidence that the use of EDIs is becoming increasingly common in routine care [15].

### Mode of injection

Potential benefits of lanreotide autogel are the ease of use due to the delivery system (i.e., pre-filled syringes) and the ability to self-administer the medication subcutaneously, which allows for safe and effective administration by HCPs, but also by the patient or a caregiver [7, 21–23]. In this real-world study, only 17.4% of the patients used the self-injection method, with most expressing a preference for HCP-administered injections. Even so, an important benefit of lanreotide autogel is that it gives patients the option to self-inject at home, if they prefer, although more training could be offered to ensure that patients feel confident in performing the injection themselves.

### Change in dose or dosing interval

Our data show that the EDI was effective in the vast majority of patients, as only eight patients (7.3%) required a change in treatment (ten treatment adjustments), most of which (70%) involved either a further extension of the dosing interval or a decrease in the dose. Only 30% (three adjustments) required a more frequent dosing interval.

### Quality of life outcomes

In general, QoL in this patient sample was consistent with previous reports [24]. However, as is to be expected in patients with acromegaly, the AcroQoL questionnaire identified several health-related issues, mostly pain and discomfort (affecting 68.5% of the sample), findings that are in line with previous reports showing that joint and musculoskeletal-related pain are present in up to 90% of patients [24]. Although pain is highly relevant to patients with acromegaly and has a clear negative impact on QoL [25], studies have shown that psychological status actually has the largest impact on QoL in this patient population [24, 26]. Anxiety and depression are common in patients with acromegaly [24] and the high prevalence of these disorders in our series (>50% of patients) likely had a negative impact on QoL. Interestingly, although long-acting SSAs provide hormonal control and improve health-related QoL [23], there is no correlation between IGF-1 levels and the subjective sense of well-being reported by patients [24, 26–29].

### Treatment satisfaction and adherence

Treatment satisfaction in our study was high, as evidenced by the mean global satisfaction score (75.1) on the TSQM-9 scale. These findings are consistent with previous reports demonstrating good patient satisfaction with lanreotide autogel [11, 29, 30]. Treatment adherence rates were excellent (94.5%), with only 6 of the 109 patients missing more than one injection. These findings are in line with other studies that have evaluated EDIs with lanreotide autogel, which have reported adherence rates ranging from 90 to 96% [9, 11]. Importantly, there were no significant differences in adherence between patients treated every 5–6 weeks and those treated every 7–8 weeks.

### Study strengths and limitations

The main limitation of this study is the observational design, with the limitations inherent to this type of study. In addition, the laboratory tests were not centralised, but rather performed in local laboratories in accordance with routine clinical practice. In addition, patients did not directly compare self-injection to HCP-injection, nor did they receive training in self-injection methods before indicating their preferences. The relatively short follow-up is another limitation, mainly because IGF-1 levels may remain normal in some patients even after discontinuation of chronic SSA therapy [31]; consequently, studies with longer follow-up are needed to confirm these findings. While the inclusion of patients treated with radiotherapy could have influenced the results, our subanalysis shows that this had no effect on the main study endpoint. Given that this was a real-world study, the inclusion of patients treated with radiotherapy (25.7% of the sample) reflects the reality of routine clinical practice, which is an important strength of this study. Given the rarity of acromegaly, the large number of patients (*n* = 109) is an important strength, as is the assessment of treatment adherence, patient satisfaction, and QoL using validated questionnaires.

## Conclusions

In this cross-sectional study, lanreotide autogel 120 mg administered at dosing intervals >4 weeks for at least 6 months was effective in controlling IGF-1 levels in more than 90% of patients. These findings provide further support for the use of EDIs with lanreotide autogel 120 mg in patients with acromegaly who have achieved good biochemical control with long-acting SSAs. Our data confirm the growing body of evidence indicating that biochemical control rates obtained with EDIs in appropriately-selected patients are equivalent to those obtained with standard dosing intervals.

## Data Availability

Where patient data can be anonymised, Ipsen will share all individual participant data that underlie the results reported in this article with qualified researchers who provide a valid research question. Study documents, such as the study protocol and clinical study report, are not always available. Proposals should be submitted to DataSharing@Ipsen.com and will be assessed by a scientific review board. Data are available beginning 6 months and ending 5 years after publication; after this time, only raw data may be available.
